# Dietary lipid is largely deposited in skin and rapidly affects insulating properties

**DOI:** 10.21203/rs.3.rs-3957002/v1

**Published:** 2024-02-22

**Authors:** Nick Riley, Ildiko Kasza, Greg Barrett-Wilt, Julian Michaud, Raghav Jain, Michaela E. Trautman, Judith A. Simcox, Chi-Liang E. Yen, Ormond A. MacDougald, Dudley W. Lamming, Caroline M. Alexander

**Affiliations:** 1 McArdle Laboratory for Cancer Research, University of Wisconsin-Madison,; 2 Biochemistry Mass Spectrometry Core,; 3 Department of Biochemistry, University of Wisconsin-Madison,; 4 Department of Medicine, University of Wisconsin-Madison,; 5 William S. Middleton Memorial Veterans Hospital, Madison,; 6 Howard Hughes Medical Institute, University of Wisconsin-Madison,; 7 Department of Nutritional Sciences, University of Wisconsin-Madison,; 8 Department of Molecular & Integrative Physiology, University of Michigan

## Abstract

Skin has been shown to be a regulatory hub for energy expenditure and metabolism: mutations of skin lipid metabolism enzymes can change the rate of thermogenesis and susceptibility to diet-induced obesity. However, little is known about the physiological basis for this function. Here we show that the thermal properties of skin are highly reactive to diet: within three days, a high fat diet reduces heat transfer through skin. In contrast, a dietary manipulation that prevents obesity accelerates energy loss through skins. We found that skin was the largest target in a mouse body for dietary fat delivery, and that fat was assimilated both by epidermis and by dermal white adipose tissue. Dietary triglyceride acyl groups persist in skin for weeks after feeding. Using multi-modal lipid profiling, we have implicated both keratinocytes and sebocytes in the altered lipids which correlate with thermal function. In response to high fat feeding, wax diesters and ceramides accumulate, and triglycerides become more saturated. In contrast, in response to the dramatic loss of adipose tissue that accompanies restriction of the branched chain amino acid isoleucine, skin becomes highly heat-permeable: skins shows limited uptake of dietary lipids and editing of wax esters, and acquires a signature of depleted signaling lipids, which include the acyl carnitines and lipid ethers. We propose that skin should be routinely included in physiological studies of lipid metabolism, given the size of the skin lipid reservoir and its adaptable functionality.

## INTRODUCTION

A growing body of research highlights the influence of altered skin function on system-wide metabolism and energy expenditure. Mice with gene mutations that specifically affect the lipid composition of their skin exhibit changes in thermoregulation, resistance to diet-induced obesity, and enhanced insulin sensitivity. While the potential role of the skin in regulating overall metabolism has been discussed in detail by us and others^1–6^, the precise mechanisms behind this interaction remain unclear. This presents a significant translational knowledge gap, as the manipulation of skin properties and cues may offer a valuable approach for improving health.

We propose that the rate of heat loss through the skin is a regulator of metabolism, since higher heat loss promotes higher rates of thermogenesis to maintain body temperature. Skin is comprised of three, closely-apposed layers are the pelt, with associated waxy sebome, the stratum corneum, assembled by epidermal keratinocytes, and the unique skin associated adipose depot, the dermal white adipose tissue (dWAT)^1,7^. The regulated properties of any of the three biomaterials could therefore be important effectors of energy expenditure.

In this study, we test whether skin function is an early responder to conditions that change skin thermal properties, and if so, which of the cell types and thermal components correlate with functional changes. We focus on the molecular lipidome, for two reasons. The first is the body of experimental data demonstrating that mutations of skin lipid metabolic enzymes confer changes of system-wide metabolism, and the second is that lipid composition is known to be modified by environmental temperature changes throughout the biological phyla, suggesting that lipid biosynthesis is an ancient target able to change thermal transfer properties as a means of adaptation^8^.

Using a short timeline of 3 days, we find that skins become more insulating in response to an obesogenic high fat diet, and more heat-permeable in response to a health-promoting diet, a diet low in the branched chain amino acid, isoleucine. Skin properties are therefore highly modifiable, and amongst the earliest functional responders to diets that affect obesogenesis and energy expenditure. Using multi-modal lipidomics, we show that the amount and specifics of the lipids from sebome and skin change in parallel with functional changes, suggesting that both sebocyte and keratinocyte could be manipulated to promote heat flux through skins.

## RESULTS

Most studies of metabolic adaptation to diet or environment use a minimum 2–3 weeks after the transition, before testing for phenotypic alterations of energy expenditure. Our goal was to evaluate whether skin adaptation preceded or followed other changes, such as an increased body weight in response to high fat feeding. We therefore chose 3 days after the diet switch, firstly to a high fat diet (**HFD**; 60% calories from fat, provided as 90% lard, 10% soybean oil, compared to control diet 18% calories from fat, provided as soybean oil).

**Fig. 1.**
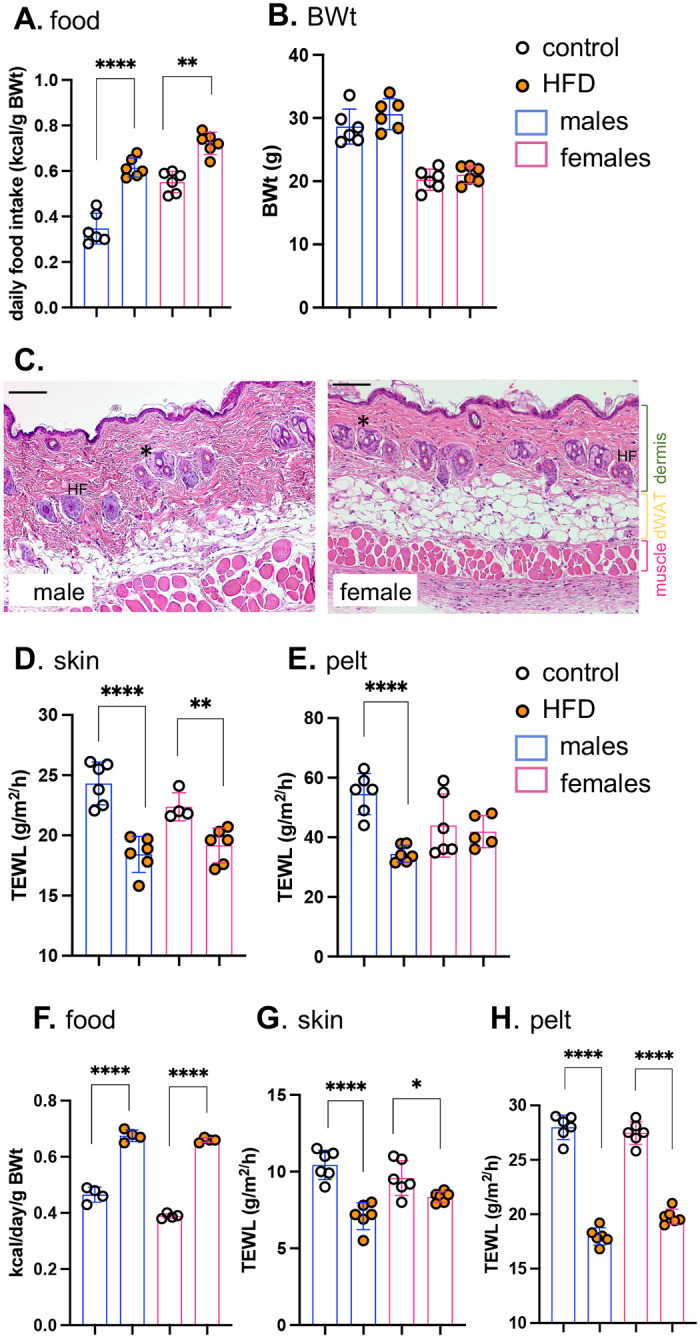
High fat diet consumption rapidly results in lower heat loss through skins. Male and female BALBc/J mice (10–15 weeks old) were switched to HFD for 3 days, and (**A**) daily food consumption assessed together with body weight (after 3 days feeding; n=6; **B**). **C**. Representative H&E-stained skin sections are shown for male and female BALB/cJ mice. Skin properties were measured by TEWL assay of skin (**D**) and pelt (**E**). Male and female C57BL/6J mice were also fed HFD and assayed for relative food consumption (**F**), and the properties of their skins (**G,H;** n=6). * p<0.05; ** p<0.01, *** p<0.001, **** p<0.0001.

We found that both male and female BALB/cJ mice ate more kilocalories (Kcal) per day when switched to HFD (Fig.1A) but within 3 days, did not yet show a significant change of body weight (Fig.1B). The average area of perigonadal white adipocytes and dermal white adipocytes was also unchanged and there were no significant changes of skin morphology, including dWAT thickness. Male and female skins are highly dimorphic, specifically male skins (mice under 6 months, fed chow) have much less dWAT than females, with thick collagenous dermis (Fig.1C).

To assess the effect of HFD on the thermal properties of skins, evaporative cooling rates were measured using rates of trans-epidermal water loss (**TEWL**). This assay is done *ex vivo* on a warm, wet heat block, to eliminate effects attributable to changing blood flow, and reported for shaved skin (dorsal “**skin**”) and **pelt** (unshaved), as described previously^2^. Heat transfer across HFD-fed mouse skins and pelts was significantly reduced, notably for males (Fig.1D,E). Obesogenesis in response to high fat diet is highly strain-dependent therefore, we also evaluated C57Bl/6J mice, a strain high susceptible to diet-induced obesity^9^. In both male and female mice, we found much reduced heat loss across skins and pelts within 3 days of high-fat feeding (Fig.1F–H).

**Fig. 2.**
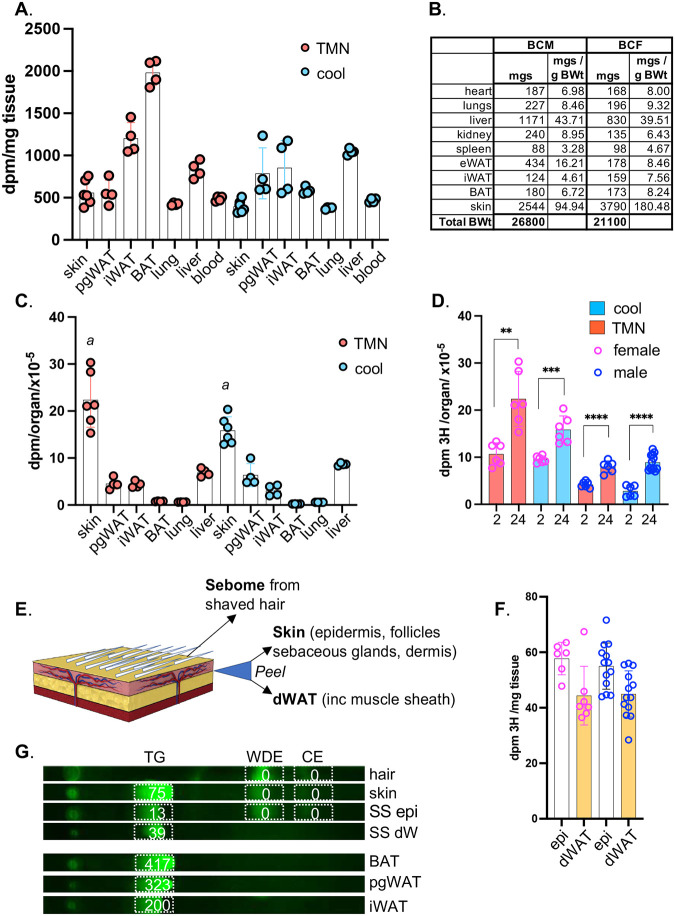
Skins assimilate more dietary acyl lipid than any other organ. BALBc/J female mice were administered 3H-triolein by gavage, and assessed 24 hours later for relative incorporation of radiolabel into the tissues indicated (pgWAT, perigonadal (visceral) WAT; iWAT, inguinal (subcutaneous) WAT; BAT, brown adipose tissue). Mice were housed either at thermoneutrality (TMN, 29°C) or cool (10°C); n≥3. **A**. Radiolabel assimilation was calculated as dpm/mg tissue for the tissues indicated. **B,C**. Calculated per organ, skins assimilated more label than any other tissue. Assimilation into iWAT was measured for the pair of iWAT depots, noting that there are 10 fat pads altogether in this class. ***a***, comparison of skins from mice housed at TMN versus cool, p=0.0001. **D**. Skins from BALB/c female and males 2 and 24 hours after radiolabel administration were assayed for their relative specific activity. **E-G**. Skins were separated into 3 fractions, **sebome** (extracted from hair), **dWAT** (separated from skin), and the skin remainder (**epi**). **F.** Epi and dWAT fractions were weighed and counted. **G.** Lipid classes were separated by thin layer chromatography (TLC**),** into triglyceride (TG), wax diester (WDE) and cholesterol ester (CE) and scraped for counting (a representative image is labeled with dpm/mg of tissue equivalent), from 3 adipose depots, along with hair, skin and separated skins (SS).

**Fig. 3.**
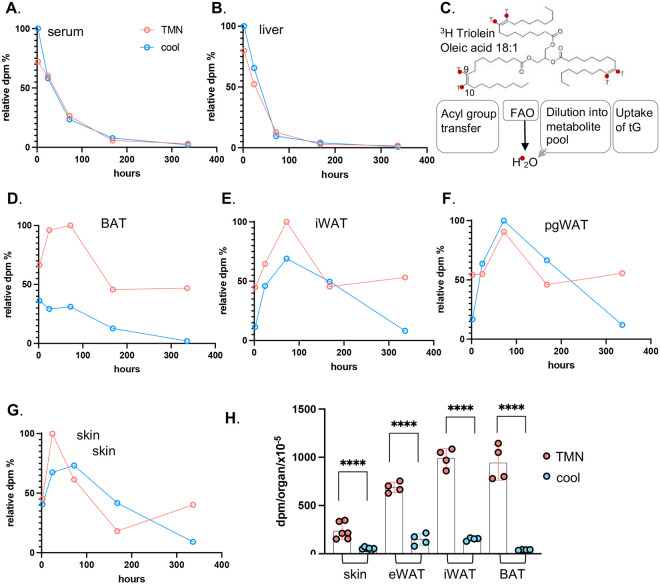
Dietary fat persists in skin and adipose depots for several weeks. **A,B.** Dilution and elimination of the radiolabel is indicated for 2 weeks post-gavage, for serum and liver. **C.** A scheme of the potential fates of ^3^H-triolein (acyl group ^3^H-label), which are not discriminated by assay of dpm/mg tissue: acyl group reshuffling, fatty acid oxidation (FAO), degradation of acyl groups and dilution into general metabolic pool, or uptake of the whole TG moiety. **D-G.** Relative specific activity of adipose depots and skin, expressed with respect to time mice housed either at thermoneutrality or in cool housing. Timepoints shown are 2h, 24h, 3 days (72h), 1 week (168h) and 2 weeks (336h) (n≥3 for each timepoint) for **H.** Statistical analysis of specific activity of tissues indicated for the 2-week timepoint. See also Fig.S2 for statistical analysis of timeline data.

At least two possibilities could explain this response, the first is that high fat consumption sends an indirect cue to skin, and the second is that incorporation of dietary fats into skins changes thermal properties. To address these possibilities, we first tested whether lipids could be delivered directly to skin from high fat diet. We used the approach to studying uptake of dietary lipids that was described by Bartelt et al^10^. We administered ^3^H-[9,10] triolein by gavage (Fig.2,3), and assessed the assimilation of radiotracer over the course of 2 weeks in serum, liver, BAT, iWAT, pgWAT and skins, for BALB/cJ males and females housed at either thermoneutrality (29°C; TMN) or at 10°C (cool).

Like other adipose depots, skins showed rapid assimilation of dietary ^3^H-triolein (Fig.2A–D and Fig.3). Normalized by tissue weight, the specific activity of skin was approximately the same as visceral adipose depots (pgWAT; Fig.2A). The well characterized thermogenic depots iWAT and BAT labeled to a 2–4x higher specific activity than other adipose when mice were housed at thermoneutrality, illustrating their role as the major metabolic drivers of thermogenesis. As expected, radiolabel was rapidly depleted from these depots in mice housed in a cool environment (Fig.2A–D and Fig.3). Skin showed a similar pattern. The specific activity of female skin was 2–3-fold higher than for males, presumably in part due to thicker dWAT (shown in Fig.1C).

When the total incorporation of ^3^H-triolein label is calculated for skins (Fig.2B), we found that skin was by far the largest target for lipid uptake (Fig.2 B,C), irrespective of environmental housing temperature. Dedicated lipid storage depots (pgWAT and iWAT) showed only 20% (each) of the total acquisition and storage capacity of skin.

To determine into the delivery site for dietary lipids into skins, we separated skins into 3 fractions (Fig.2E). These reflect the three distinct lipid-based thermal barriers in skin: the pelt/hair, coated in a sebome of mostly wax diesters; the stratum corneum, a proteo-lipid cross-linked sheath of enucleated keratinocytes providing the top layer of the epidermis; and the dermal white adipose tissue (dWAT) underlying the pelt/epidermis/dermal layers (see Fig.6). Using enzymatic dissociation, we separated the dWAT from the dermal/epidermal layer (epi) of shaved skins to generate a highly enriched dWAT adipose fraction. These fractions (epi and dWAT) were labeled to approximately the same specific activity; 60% recovery from epidermal fraction, and 40% from dermal adipose (Fig.2F). Thus ^3^H oleate was acquired by the keratinocyte/sebocyte-enriched tissue fraction to a similar extent as the adipose depot. Label accumulation by hair was low/undetectable over the 2-week period.

To determine which lipid class had acquired ^3^H oleate, we separated the lipids in sebome, skin, and separated skins (SS) by thin layer chromatography (TLC) and found that the triglyceride fraction of skin accounted for all detectable counts (Fig.2G). There were no counts in the wax diester (WDE) or cholesterol ester (CE) fraction. Using this method, the TG fraction of BAT yielded the highest specific activity, as expected.

Predictably, the specific activity of sera and liver diluted out rapidly within 4 days (96 hours; Fig.3 A,B, **S2**). The specific activity measured (dpm/mg tissue) represents an equilibrium between uptake of ^3^H-triolein triglyceride or ^3^H-oleate acyl groups versus oxidation of fatty acids or their dilution into the general pool of metabolites (Fig.3C). We assume that only acyl group transfer is effectively scored by this labeling strategy.

In contrast to liver and sera, the specific activity of adipose depots and skin did not dilute out rapidly, in fact the specific activity was maintained to 50% of maximum specific activity even after 2 weeks of gavage (Fig.3D–H), as long as thermogenic lipid oxidation was suppressed. For mice housed cool, the radiotracer was depleted within 2 weeks, demonstrating that skin-associated lipids were also contributing to thermogenesis. We considered whether the gavage lipid dose was higher than usual and could be accumulating artifactually in skins. However, the dose administered was only 50% of the lipid intake present in a daily ration of chow.

We conclude that the rapid and sustained uptake and storage of ^3^H-oleate in skins is consistent with a direct delivery mechanism from diet. As an alternative approach to demonstrating that skin-associated lipids reflect dietary lipids directly, we fed mice with a Western diet (**WD**), enriched in the medium chain fatty acids (12:0,12:1,13:0, 13:1,14:0,14:1,15:0; **MCFA**s) typical of milk fat^11^. These MCFAs are absent from our standard chow, in which soybean oil (60%) and grains are the fat sources.

**Fig. 4.**
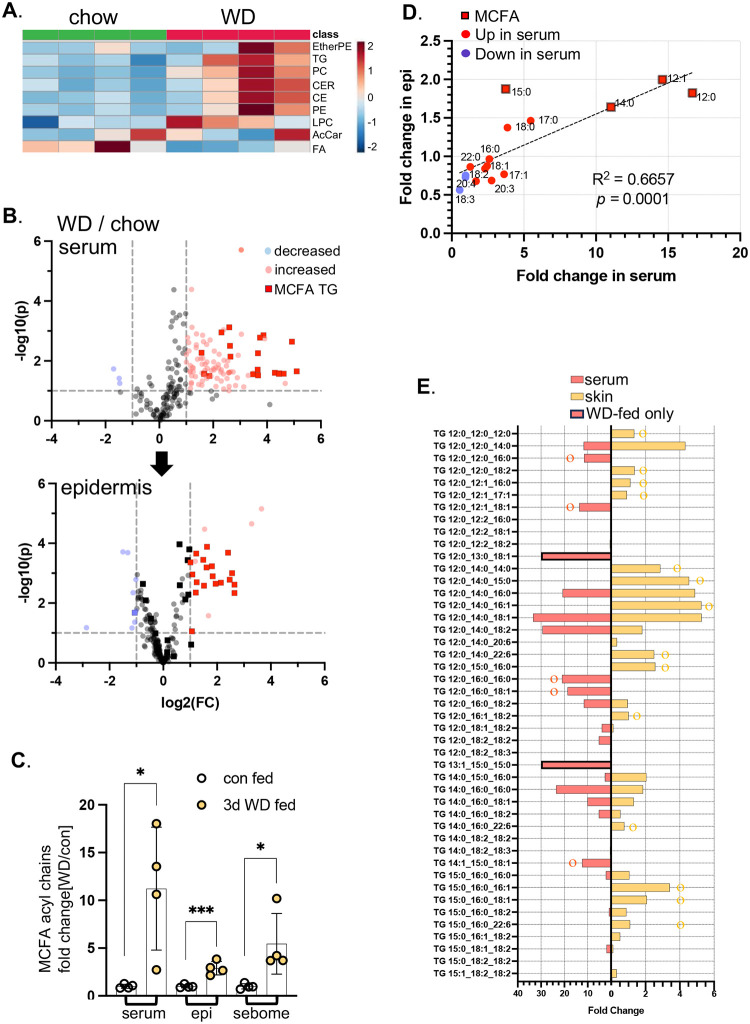
Milk fat acyl chains are delivered to skin-associated triglycerides. **A.** Feeding of Western diet for 3 days induced typical changes of circulating lipid classes, shown here for 4 mice in each group (WD or chow-fed). Data is shown as an unclustered heatmap analysis of sera samples (EtherPE, ether phosphatidylethanolamines; PC, phosphatidylcholines; Cer, ceramides; CE cholesterol esters; PE, phosphatidylethanolamines; LPC, lysophosphatidylethanolamines; AcCar, acyl carnitines; FA, free fatty acids; n=4). **B.** Volcano plots of the circulating lipidome and epidermis, showing significantly changed lipids (WD/chow), including TGs with medium chain fatty acids (MCFA), a signature of Western diet consumption. **C.** Fold change of MCFA acyl chain scores were compared for the tissues indicated. **D**. Amounts of MCFA-TGs were expressed as a correlation plot for serum versus skin. **E.** Fully specified MCFA-containing TGs were compared for serum and for skin, to test for a direct correlation. Species marked with o or o are unique to either serum or skin (respectively) or are present only in serum (WD-fed only).

Sera from mice fed WD for 3 days showed increases in several classes of circulating lipid, as reported in many previous studies (Fig.4A), including ceramides (CER), triglycerides (TG), phosphatidylcholines (PC), cholesterol esters (CE) other phospholipids. Using LS/Q-TOF mass spectrometry, we identified TG species containing MCFAs that became enriched after feeding WD, in sera and epidermis (Fig.4B). The total amount of MCFA acyl chains increased in serum, epidermis and sebome (Fig.4C), and there was a strong correlation between serum [MCFA] and skin [MCFA] (Fig.4D). There was a trend to increased MCFA assimilation in dWAT (**Fig.S3**).

We considered whether dietary TG could be delivered intact to skin, seeking direct matches between increased circulating TGs with those observed in epidermis (skin; Fig.4E). We found many examples of MCFA-containing TGs which were increased in skin, but were not present, or did not increase in the serum of WD-fed mice. We propose that this implicates a lipoprotein-lipase (LPL)-mediated acyl group extraction of circulating TGs containing MCFAs, with specific re-assembly of TGs by the skin epithelial cell population. This processing is likely to represent a rate determining step for the assimilation of TGs by skin.

Given that our data show increased insulative properties of skins after high fat-diet feeding, we turned to ask how skins might respond to health-promoting diet interventions. We and others have shown that dietary protein restriction reduces fat mass and adiposity in both mice and humans^12–14^. We have shown that these effects are mediated by the three branched-chain amino acids (BCAAs), and restriction of all three BCAAs or isoleucine alone can rapidly restore leanness to male mice with diet-induced obesity (DIO), associated with increased energy expenditure and better glycemic control^12,15^.

**Fig. 5.**
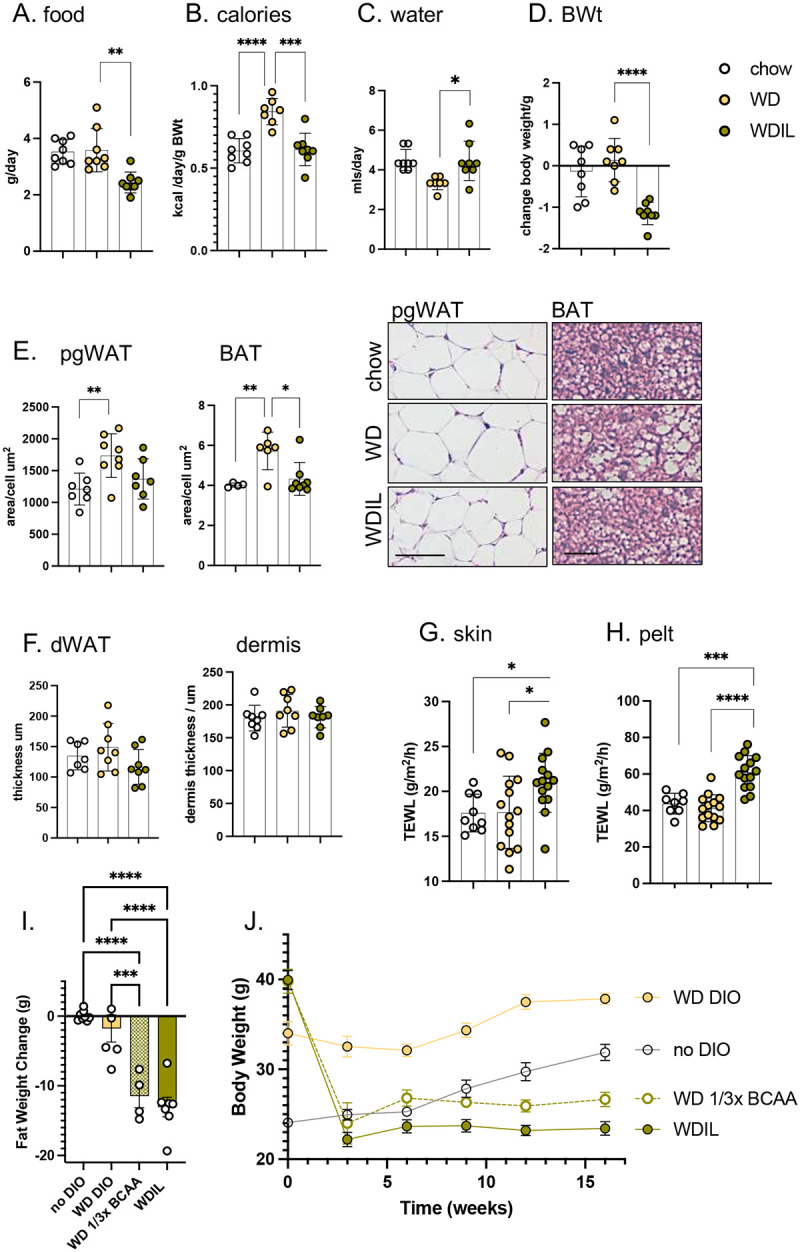

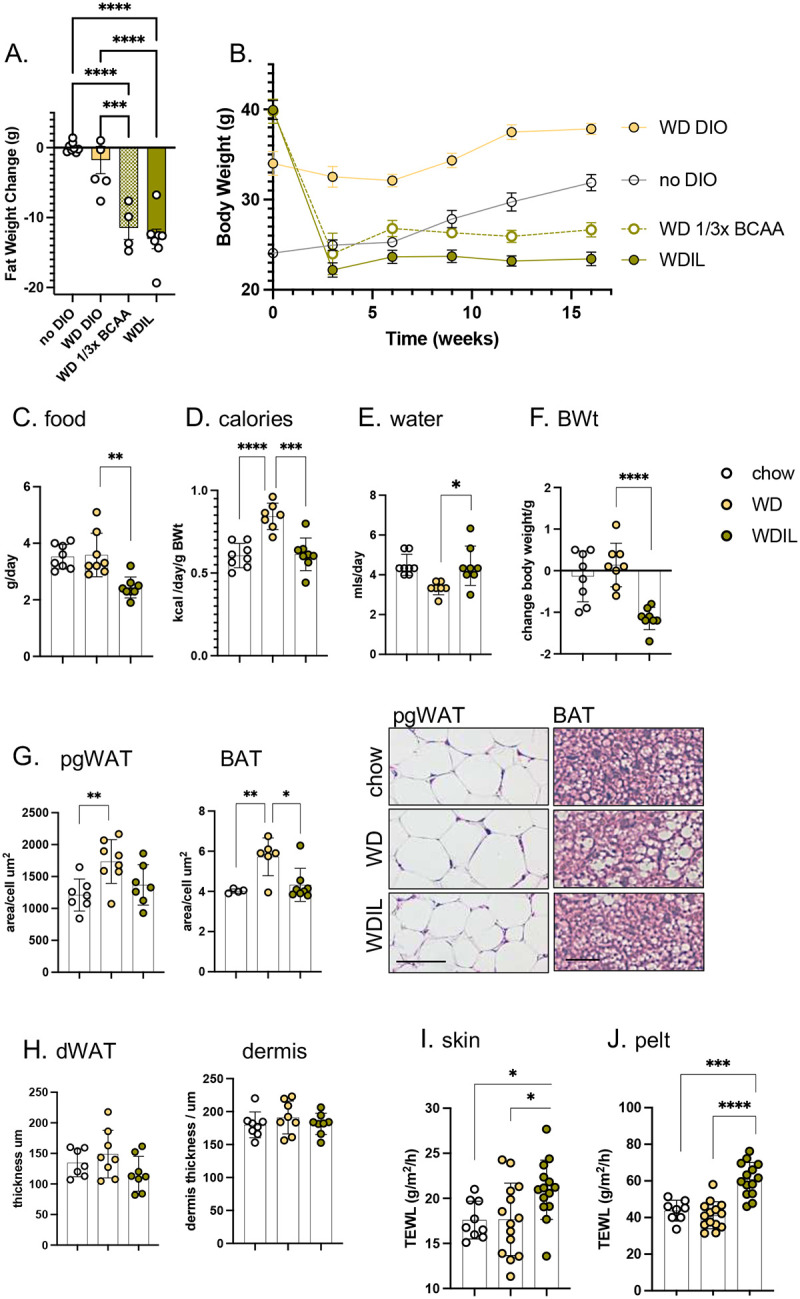
Mice fed a low-isoleucine Western diet rapidly develop heat-permeable skins. Female C57BL/6J mice (10–15 weeks old) were switched to Western diet (**WD**) or low-isoleucine Western diet (**WDIL)** for 3 days, and daily water and food consumption assessed, either as grams/day or kcal/day/g body weight (**A-C,** n=7–8). **D**. Body weights were measured after 3 days**. E, F.** Changes to adipocyte depot lipid loads were calculated as the average area of pgWAT adipocytes or the lipid droplet area/cell for BAT, shown also as representative H&E-stained skin sections. Aspects of skin form and function are shown as dWAT and dermal thickness (**F**), and heat permeability of skin (**G**) and pelt (**H**). **I,J.** To illustrate longer term impact of WDIL diet, C57BL/6 mice were made obese by 16 weeks of WD feeding (**WD DIO;** TD.88137) or maintained at regular weight using a regular fat content diet (**no DIO;** TD200693). Mice from the DIO cohort were then switched to low isoleucine (**WDIL**; TD.200692), low branched chain amino acid (**WD 1/3xBCAA**; TD.200691), or amino acid-defined WD (**WD DIO**; TD.200690) for 3 weeks (I), or longer (J).

To demonstrate the effect of isoleucine restriction in female mice, we switched the diet of C57BL/6 female mice made obese by 16 weeks of WD consumption to a diet with low isoleucine (**WDIL**) or low branched chain amino acids (leucine, isoleucine and valine, all at 33% of standard amounts, **WD 1/3xBCAA**; Fig.5A,B; **S4)**. The diets all contain identical amounts and sources of fat and sugar; the calories removed through reductions of BCAAs are replaced with non-essential amino acids to maintain the diets as isocaloric (**Table S1**). We found that mice rapidly lost weight on WDIL diet without a reduction in calorie intake (Fig. 5B**, S4**). Analysis of body composition showed that the weight reduction in mice fed the BCAA and Ile-restricted diets primarily resulted from reduced fat mass (Fig. 5A**, S4**).

To test whether loss of heat through skins could contribute to the rapid weight loss induced by this low-isoleucine diet, skin function was assessed for C57BL/6 female mice fed WD or WDIL for 3 days. Over this short timeline, mice fed WDIL adjusted their calorie intake to match chow-fed animals, where mice fed WD consumed 40% more calories (Fig.5C,D). Mice eating WDIL did not show the suppression of water intake induced by WD consumption (Fig.5E). After only 3 days of feeding, mice eating WDIL lost over 1 gram of body weight (Fig.5F). Mice eating Western diet showed no significant body weight increase at this time point, however, pgWAT adipocytes were already significantly larger, and BAT adipocytes contained a higher lipid load (Fig.5G). In this context, the permeability of skins and pelts were increased by 25% and 40% respectively (Fig.5I,J).

Note that skins from mice fed WD are unlike HFD-fed mice in showing no net change in skin properties after 3 days feeding. Calories from fat are 60% for HFD, 42% for WD and 18% for chow. However, WD combines high sucrose (34% w/w) with elevated fat content, and it is this combination that has been shown to be highly proinflammatory, both in general and for skin specifically^16,17^. It is perhaps not surprising that the thermal phenotype induced by WD is complex.

**Fig. 6.**
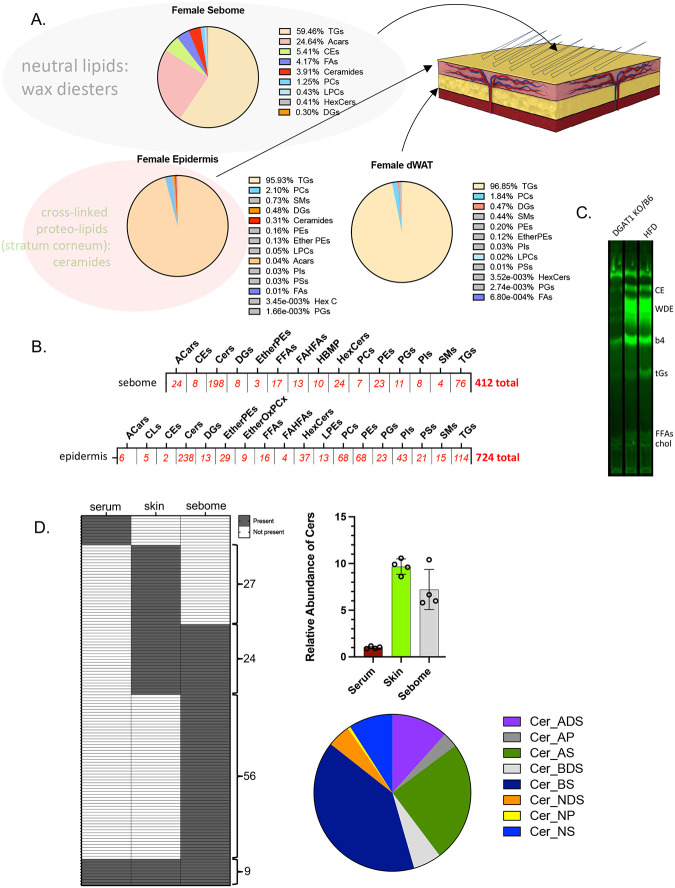
Multi-modal lipid analysis of skins. Skin tissue fractions produced as described in Fig.2 and Materials and Methods were analyzed for their lipid composition using LC/Q-TOF mass spectrometry. Relative quantities, types and lipid species number identified and screened for the skins analyzed for this study are shown (**A,B**). The larger grey circle shown around the sebome indicates the neutral lipid wax esters (and potentially other classes) which are not ionized and identified by mass spectrometry; these are quantified and analyzed instead using thin layer chromatography (TLC) and FAME derivatization (**C**). The larger pink circle around the epidermal lipids indicates the cross-linked proteolipid sheath called the stratum corneum, highly enriched with ceramides, and invisible to our analyses. **D.** Analysis of ceramide species present in serum, skin and sebome show relative amounts (per mg of tissue), ceramide classes identified, and exact species present in each tissue type (named in **Fig.S6**). The ceramide species shown in (D) were identified in positive mode of lipid mass spectrometry; the totals shown in (B) are identified in both positive and negative modes.

We turned to molecular lipidomics to identify molecular correlates of altered thermal barriers. Each of the three biomaterials comprising the skin thermal barrier depend upon different processes of lipid biosynthesis for their properties (Fig.6A,B). Specifically, TGs comprise most of the lipids of dermal WAT; the sebome is synthesized by sebocytes (embedded in the epidermis) and comprises mostly wax diesters (Fig.6C). The epidermal stratum corneum is made by keratinocytes and comprises a cross-linked proteolipid matrix with mostly ceramides. We applied a MALDI-TOF untargeted lipidomics pipeline in both positive and negative modes, to identify and quantify nearly 1200 individual lipid species (Fig.6B). We assume that neutral lipid species may be primarily responsible for thermal properties: these species are not captured by MALDI-TOF, but can be identified by derivatization of lipid classes separated by TLC (Fig.6C). One notable exception to the lipids assayed using this multi-modal approach is cholesterol, which is a major component of the stratum corneum^18,19^.

The ceramides present in serum, skin and sebome are almost exclusive (Fig.6D**,S5**); this likely reflects their different functions, as signaling molecules, precursors to the cross-linked ceramide sheath encasing mammals, and components of the thermal barrier coating mouse hair, respectively. The most abundant ceramide species in sebome are ahydroxy-dihydroceramides (Cer_ADS), accounting for 75% of total. The majority of skin-associated ceramides are non-hydroxy sphingosines (Cer_NS), which are detected in only trace amounts in sebome^20,21^. Thus, the key ceramide synthase activities are likely to be distinct.

**Fig. 7.**
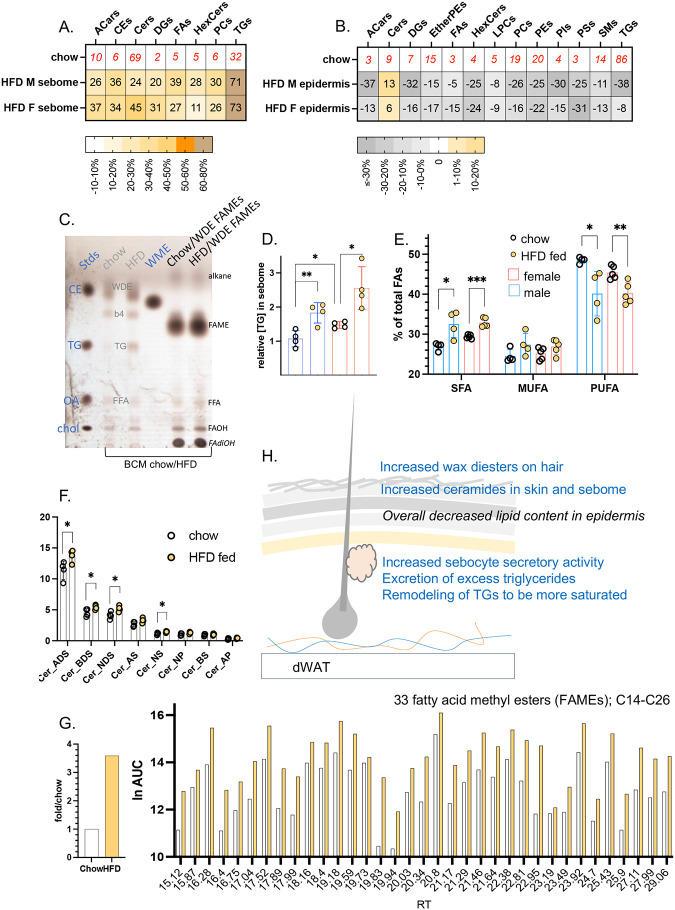
High fat diet consumption results in changes to known thermally active components of the sebome and skin lipidomes. **A,B.** The relative abundance of each lipid class is shown for BALBc/J male and female sebome and epidermis (n=4; additional lipid abbreviations noted here are DGs, diacylglycerols; HexCers, hexaceramides; PIs, phosphoinositides; PSs, phosphoserines; SMs, sphingomyelins). **C.** Analytical TLC of lipid extracts from hair of mice fed control or HFD diets for 3 days (pooled from 3 mice, repeated twice on separate samples). The lipid mix directly extracted from hair is shown labeled in grey, the WDE fraction after base-esterification to derivatize acyl-fatty acid methyl esters (FAMEs) are shown labeled in black. WDE, wax diester; b4, unidentified band 4; tentative identification motility of alkanes and fatty dihydroxy alcohols (FAdiOH). Other lipids are identified by co-migration with standards. **D**. The increase in TGs present in sebome from male and female BALBc/J mice fed HFD are quantified, together with the relative acyl chain composition of the TGs (**E**). **F.** Consistent increases of each of the top 5 ceramide classes in sebome of HFD-fed mice. **G**. Quantitation and enumeration of 33 FAME species derived from sebome wax diesters, observed in the resolution range of the GC/MS column (C14-C26), and reported as retention times (RT). Identification of 13 of the FAME species was done by comparison with a standard mix (**Fig.S1**). **H.** Summary scheme of the changes of sebome and epidermis observed in response to HFD feeding.

Using separated skins from mice fed HFD for 3 days, we analyzed the lipid fraction of BALB/cJ male and female hair-associated sebome and epidermis. Both epidermis and sebome were significantly changed by high fat consumption, characterized as a widespread depletion of approximately 20% of all lipids in the epidermis, and a corresponding gain of lipids secreted onto the pelt (sebome; Fig.7A–B). However, we evaluated whether the lipids appearing in sebome were the same lipids becoming depleted in epidermis and found no support for this hypothesis. This suggests that lipid depletion is associated with altered metabolic function in keratinocytes, probably fueling the remodeling of barrier ceramide biosynthesis^18^.

The TGs present/mg hair increased by 70% (both male and female) and were distinguished from the changes in other lipid classes by this larger accumulation (Fig.7A,C–E). Furthermore, saturated TG acyl chain became highly enriched, whilst poly-unsaturated acyl chains became depleted (Fig.7E). Thus, Males fed HFD show an increase of 5.5% saturated fatty acids (from 27% total in control fed condition; SFAs), and a decrease of 8.4% (from 48.5% total; PUFAs), where females show a similar but muted trend. Given that increased acyl chain saturation changes the melt temperature and physical properties of TGs, this may contribute to significant functional change.

For the epidermis, the only lipid class that did not become depleted were the ceramides. The mass spectrometric assay measures only the soluble ceramide pool, intermediate to the assembly of the highly insoluble and cross-linked stratum corneum. Likewise, the ceramides present in the sebome increased by approximately 20% across all 8 classes identified, thus the amount changed but the composition remained the same (Fig.7F).

Surprisingly, even the wax diesters coating the pelt hairs were affected by short exposure to HFD diet consumption. Although hair goes through a month-long asynchronous process of growth and involution^22^, we conclude that the wax coating is labile. Thus, WDEs accumulate over 3x on the coats of mice fed HFD (Fig. 7G). The changes of ceramide amount were higher for males than females, a pattern that correlated with the exacerbated pelt insulation for males fed HFD (Fig.1). Indeed, the baseline amounts of ceramides in females was >2-fold higher.

The changes we observed in skins of mice fed HFD are summarized in the scheme of Fig.7H: there were increased wax diesters on hair, increased ceramides in skin, increased sebocyte activity, with excretion of excess triglycerides and remodeling of the secreted triglycerides to be more saturated.

**Fig. 8.**
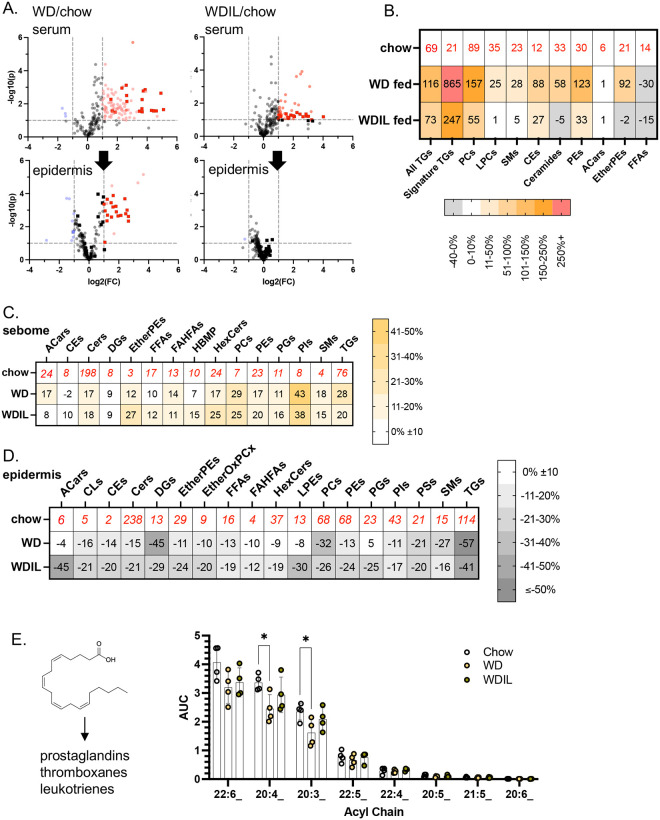
Low isoleucine Western diet prevents dietary delivery of acyl chains and mobilization of proinflammatory oxylipids from the epidermal reservoir. **A.** Relative appearance of Western-diet enriched MCFAs in epidermis of WDIL-fed mice compared to WD-fed mice (see Fig.4; n=4). **B.** Heatmap of changes induced in circulating lipidome for WD- and WDIL-fed mice. **C,D.** The relative abundance of each lipid class is shown for C57BL/6J male sebome and epidermis, for control, WD and WDIL-fed mice. **E.** Oxylipid acyl chains, precursors of inflammatory cytokines (shown left hand side) were quantified for epidermal fractions from mice in all 3 diet conditions.

By way of contrast, we analyzed the hyper heat-permeable skins from mice fed WDIL using the same techniques. We found that WDIL-fed mice partly resisted the increase in MCFA-containing circulating lipids that accompanies digestion of WD, and likewise, resisted delivery and accumulation of MCFAs in skins (Fig.8A). More detailed analysis of the changes in sera of WDIL-fed mice showed that several lipid classes were profoundly affected, including the phosphatidylcholines (and associated lysophosphatidylcholines), ceramides, sphingomyelins, and cholesterol esters (Fig.8B). We conclude that low isoleucine-containing diet consumption leads to a profound alteration of the digestion and processing of WD.

Similar to HFD-feeding, skins from WD-fed mice showed a broadly similar trend towards sebome lipid elevation with corresponding depletion of total lipids from epidermis. However, unlike HFD consumption, the relative secretion of TGs was not increased above all the other lipid classes, therefore excess fat does not vent out through the skins of WD-fed mice to the same degree (Fig.8C). Despite parallels with the results of HFD-feeding, there were distinctive changes in the depletion of specific lipids from epidermis (Fig.8D). The first was that the total amount of the oxylipid acyl chain reservoir stored in TGs was significantly reduced (25%), notably 20:4 and 20:3 (Fig.8E). These oxylipids can serve as precursors to inflammatory lipid classes. This mobilization of acyl chains from the TG reservoir was absent in WDIL-fed mice.

**Fig. 9.**
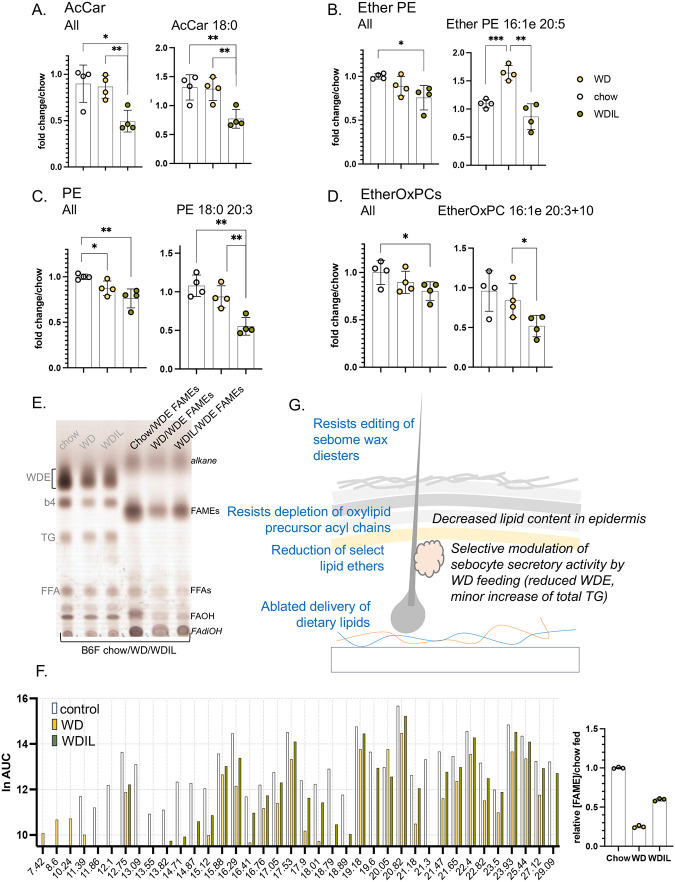
Summary of changes of heat-permeable skins induced by feeding low isoleucine diet. **A.** Several classes of lipids showed changes specific to low isoleucine-fed mice (**A-D**, n=4). **E.** Quantitation of WDEs by TLC for all 3 diet conditions (annotated as for Fig. 7); shown is a representative TLC (pooled extracts from 3 mice, repeated twice with separated mice) and quantitation of WDE-derived FAMEs (prepared as for Fig.7G). G. Summary scheme of the changes of sebome and epidermis observed in response to WD/WDIL feeding.

The second trend noticed in skins from WDIL-fed mice were changes to some key lipid classes most often cast as signaling lipids. These include acyl carnitines (AcCar), ether-linked phosphatidylethanolamines (Ether PEs), and related species such as phosphatidylethanolamines (PE) and ether-linked oxidized phosphatidylcholines (EtherOxPCs; Fig.9A–D). Acyl carnitines were significantly depleted from WDIL-fed skins, but unchanged in WD- or chow-fed samples; thus this change is a unique gain of function for the WDIL-fed mice. Similar changes were noted for PEs, Ether PEs and EtherOxPCs. Surprisingly, and in contrast to the response to HFD-feeding, WD-feeding reduced the amount of hair-associated wax diesters by nearly 80% (Fig.9E,F), and mice fed WDIL-diet resisted this phenotype (Fig.9E,F).

## DISCUSSION

More than other adipose depots and specialized lipid metabolism tissues such as liver and muscle, we have shown that skin is the dominant destination for triglyceride acyl chains. Indeed, skin can store dietary fats for weeks after consumption, and is one of the depots that shows a higher flux of lipid stores in mice when thermogenesis is active. Skin therefore resembles other adipose depots where the half-life of triglycerides has been characterized. For example, in humans, the triglycerides present in adipocytes were estimated to be turned over just six times during the average 10-year life span of an adipocyte^23,24^. This turnover was regulated by β-adrenergic, insulin and other endocrine factors. We have shown previously that dWAT is not susceptible to β-adrenergic-induced lipolysis^25^, and indeed little is known about when and how the lipid stores of this dominant tissue are mobilized. A report from Horsley and colleagues showed that skin wound healing was likely fueled by proximal lipids released from dWAT which promoted macrophage inflammation and initiated repair^26^. We speculate that skin may have an important role in the uptake of other circulating metabolites, including other lipids and glucose. Although the regulation of skin-associated fat is different for human^25^, this depot is large and metabolically active. Indeed, for lean women, this can be the largest adipose depot, varying between 6.5 −15 mm thick for individuals (an average of 15.8 kG) in a manner unrelated to general adiposity. Its regulation could be key to understanding personal energetics.

We show here that diet can induce rapid changes in the insulating properties of skin: thus within only 3 days, high fat diet reduces heat lost by evaporative cooling by up to 28%, as measured for skins with or without hair from both BALB/cJ and C57BL/6J mice. The consumption of high fat diet promotes insulation, suggesting that this could exacerbate the obesogenic effects of high fat consumption by reducing the balance of thermogenesis.

At this early timepoint, skins show no significant changes in structure, as measured from histology. Although the dWAT layer was only beginning to thicken after 3 days, there were already molecular changes in both the sebome and epidermis. In particular, there were several changes in the molecular composition of the thermal biomaterials which would be consistent with prior studies of relative cold stress of mice with mutations in lipid metabolism. For example, there were increased pools of the precursor ceramides of the stratum corneum, suggesting that production of stratum corneum is increased. Distinct classes of ceramides are arranged as highly ordered structures, with distinct physical states with different properties^27^.

More novel are the changes we observed in the neutral wax diester classes which coat the skin and hair. Although hair only renews approximately monthly in an asynchronous pattern of replacement^1,28^, the wax diester coating was increased by >3-fold. We also note that sebome secretion provides a vent for excess dietary triglycerides, in this case increasing by 70%. However, this is not a predominant means of lipid excretion compared to the other abundant skin-associated lipid classes (see Fig.6): indeed, we estimate that the whole pelt of a chow-fed mouse contains approximately 0.43 mg of triglyceride, compared to 0.74 mgs for a mouse fed HFD (assuming a total 200 mgs hair per pelt). A pattern of triglyceride venting through sebome was suggested by Choa et al to be a major route for excess triglyceride secretion induced by the cytokine thymic stromal lymphopoietin (tslp)^29^. Although unlikely to account for the approximately 15g of adipose-associated triglycerides lost during the 3 weeks of this study, sebome-associated triglyceride levels could be an important biomarker for circulating triglyceride levels and/or changing energetics. Interestingly, over-production of skin TGs was also noted after exposure to *acnes* bacteria^30^. Furthermore, the ceramide component of stratum corneum is continuously shed: these species are biosynthesized from triglyceride stores assembled by keratinocytes^18^, and therefore this too could provide a means to excretion of excess dietary lipid.

Both Western diet and high fat diet induced enhanced lipid secretion into sebome, along with depletion of lipid stores in epidermis, perhaps fueling the synthesis of ω-(O)-acylceramides. We speculate that there is a systemic cue induced by dietary fat consumption that activates lipid metabolism and excretion through the sebome, perhaps mediated by the functional communication axis between gut and skin^31^. For example, immediately upon feeding of high fat diet, there is a functional adaptation of intestinal epithelial cells along a timeline corresponding to this study^32^. Sebocytes comprise some 2% of body mass, and are highly connected to neural and endocrine networks^33^; they have a high energy requirement, and their lipid products are important not only for hair shaft eruption but provide a signaling role to the skin cell community, providing feedback regulation of their own activity^34^.

The cocktail of sugar and milk fats comprising rodent Western diet is famously inflammatory compared to a diet with high fat alone^35^; it is widely accepted that the cocktail is largely responsible for the pathologic impact of Western diet. This was specifically noted for skin by several groups, assessing skin immune function specifically for Western diet-fed mice compared to mice fed high fat or high sugar^17,36,37^. These studies showed that Western diet feeding exacerbated susceptibility to the TLR7 agonist imiquimod, leading to the development of psoriasiform dermatitis. A study of the molecular basis of balding induced by fish oil consumption revealed a clear direct relationship between dietary lipids, skin immune cells and their specific recruitment to the hair follicle^38^.

Interestingly, estimates derived from mass spectrometry suggest that at least 2.5 μgs of oxylipid acyl chains are typically immobilized in the triglyceride fraction of epidermis, of which 25% are released after consumption of Western diet. The predominant oxylipid acyl chains are 22:6, 20:4 and 20:3, all precursors of the inflammatory lipokines (prostaglandins, leukotrienes and thromboxanes). It is tempting to draw a conclusion that this mobilization provides the substrates for the triggered inflammatory reaction characteristic of Western diet fed mouse skins.

By way of comparison with the hyper-insulating effects of a high fat diet, the skin from mice fed a Western diet with low isoleucine became more heat permeable. This functional change was observed at a time when body weight had decreased by about 1 gram, which we have previously shown to be the new, lean, steady state for these mice^12,15^. This is therefore consistent with our hypothesis that the skins of mice with higher energy expenditure associated with increased thermogenesis are modified to allow the exit of more heat.

Since our data suggests that dietary lipids are delivered to skins via sera, we evaluated sera from mice fed Western diet with low isoleucine content, and found that low isoleucine diet prevents the majority of seraassociated changes. Specifically, WDIL-fed mice resist the elevation of phosphatidylcholines, sphingomyelins and ceramides. A defect of dietary processing and distribution was confirmed by absent MCFA enrichment in skins after consumption of the milk fats typical of Western diet. Unlike high fat diet, Western diet does not promote the accumulation of epidermal ceramide precursors, or sebome-based wax diesters, which is likely to explain why Western diet-fed mice do not show a net change of heat transfer across skins. However, amongst the changes induced by Western diet feeding, skins show selective phenotypes associated with reduced dietary isoleucine. Not only do WDIL-fed mice resist the changes induced by WD consumption, there are decreased amounts of lipids with signaling roles, including acyl carnitines, phosphatidylethanolamines and ether lipids such as ether phosphatidylethanolamines^39–41^. We suggest that these lipids may be associated with a particular cell population depleted by low isoleucine feeding, possibly immune cell populations.

Overall, we conclude that dietary lipids are taken up and stored by skins and alter the properties of those skins to modulate energetics. We have shown that a “health promoting” diet is associated with increased heat loss through skins, whereas an obesogenic diet is insulating. Testing of other dietary paradigms will show how widespread this phenomenon is. Diet could modify the thermal properties of skins either directly, by incorporation into thermally-active lipids, or indirectly, via signaling to cells of the skin. The molecular changes documented here implicate diet as a modulator of both sebocyte and epidermal keratinocyte functions, where each is responsible for a different component of the thermal barrier.

## MATERIALS AND METHODS

### Ethical Approval. Mice.

These studies were performed in strict accordance with the recommendations in the Guide for the Care and Use of Laboratory Animals of the National Institutes of Health. Experimental protocols were approved by the University of Wisconsin School of Medicine and Public Health Institutional Animal Care and Use Committee (IACUC) and the William S. Middleton Memorial Veterans Hospital IACUC. The number of mice used to perform this study was minimized, and every effort was made to reduce the chance of pain or suffering. All authors understand the ethical principles and confirm that this work complies with the animal ethics checklist.

### Mice.

Mouse strains and sexes are specified in the results and figure legends: C57BL/6J (Jackson labs cat#00664) and BALB/cJ (Jackson labs cat#00651). For routine housing, mice were housed at constant temperature (19–23°C) in 12 h light/dark cycles with free access to water. The diets used were standard chow (Harlan Teklad Global Diet 2018), high fat diet (HFD; Envigo diet# TD.06414, 60% calories from fat); amino aciddefined Western diet (41% calories from fat, 21% w/w milk fat, 34% sucrose, Envigo diet# TD.160186) and a matching diet with low isoleucine (67% reduced (0.254%) isoleucine, Envigo diet# TD.170484), as described by Lamming and colleagues^12,15^. At the start of all diet switch experiments, mice were weight-matched and randomized to diet group. To demonstrate the long-term impact of low isoleucine diet consumption on mice with diet-induced obesity (DIO), C57BL/6J female mice were pre-conditioned by feeding a Western diet for 16 weeks (TD.88137), where the non-DIO cohort remained on a control diet (no DIO; TD.200693). Then WD-fed mice were divided between amino acid defined (AAD) Western diets containing low branched chain amino acids (WD 1/3x BCAA WD; TD.200691), only low isoleucine (WDIL; TD.200692), or standard amino acids (WD DIO; TD.200690). Full diet descriptions, compositions and item numbers are provided in Supplemental **Table 1**.

### Non-standard housing and metabolic phenotyping.

For experiments with non-standard housing temperatures, mice were singly housed in environmental chambers (Memmert HPP750 or Caron 7350). Temperatures used were either thermoneutrality (29°C) or cool (10°C), as described previously^42^. For longer term experiments, were weighed weekly and body composition determined using an EchoMRI Body Composition Analyzer.

### Histological Analysis.

Skin, BAT, perigonadal WAT (pgWAT) and the inguinal (mammary) subcutaneous fat pads (iWAT) were dissected for histological processing as described in Kasza et al^25^. Briefly, paraformaldehydefixed, paraffin-embedded samples were H&E stained and assayed as follows: 1) dWAT thickness: 6 images of H&E-stained, non-anagen fields of skins (equivalent to ≥4500 μm linear dWAT) were assayed by image analysis (dividing total area by length). 2) Assay of BAT lipid stores: Lipid droplets were identified in gray scale images using circularity (0.1–1.0) and diameter (0.1–50 μm) thresholds in 6 independent fields of BAT (>1200 cells) and quantified using the open-source Fiji image processing package (https://loci.wisc.edu/software/fiji); data is expressed as average lipid droplet size (μm^2^). 3) pgWAT adipocyte size assay: 3–6 images across the length of the fat pad were scored for adipocyte size (measured as number of adipocytes/area scored) in each of 4–6 mice (total adipocytes scored >1500/mouse).

### Radiotracer administration, assay and skin dissociation.

Mice were acclimated to thermoneutrality before administration of Triolein [9,10–3H(N)] (ARC cat#0199), diluted 1:10 in canola oil. Mice were administered 10 μCi / 100 μl by gavage, and rehoused into controlled temperature housing, as indicated. Mice were euthanized and tissues dissolved in Solvable (Perkin Elmer cat#6NE9100) according to the manufacturer’s instructions, diluted into UltimaGold for liquid scintillation counting. Hair samples were collected for sebome analysis. For the preparation of separated skin fractions, skins were floated dermis side down onto cold elastase/DMEM/Hepes in a 6-well tissue culture dish for 6 hours. Tissue dissociation grade elastase (Sigma cat#E1250) was diluted to 0.12 mg/ml into DMEM (Gibco, high glucose cat#11965118) / 20 mM Hepes pH 7.4. Skins were rinsed in PBS, blotted, turned epidermis down and the dWAT layer scraped off manually with forceps.

### Thin layer chromatography (TLC).

Lipids were extracted from hair (up to 50 mg) sequentially, using firstly 2:1 chloroform: methanol, and then acetone (4 mls each). Extracts were inverted to mix, incubated at room temperature, the extract decanted, combined and dried down. Lipids were resuspended in 4:1 chloroform:methanol, and loaded onto preparative TLC plates (Supelco TLC silica gel-60 glass plates; Millipore Sigma cat#1.00390.0001), and separated through 3 phases (as described by Choa et al^29^). The first phase was 80:20:1 hexane:isopropyl diether:acetic acid, to 50% plate height, second phase was 1:1 hexane:benzene to 80% plate height, and third phase was hexane, to 90% height. Lipids were visualized either using primuline (0.005% in 80:20 acetone:water)^10^ or charring with cupric sulfate (sprayed with 10% cupric sulfate in 8% phosphoric acid, dried, and baked at 300°F for 10 minutes).

### Fatty acid methyl ester (FAME) analysis.

Lipids were scraped from TLC plates of separated lipids from 3 mice, and all analyses were performed in duplicate. Lipids were dissolved in toluene (100μl), and processed to fatty acid methyl esters by base-catalysed transesterification (Lipidmaps.org). Thus, 200μl of sodium methoxide (FisherSci cat#AC427228000) were added, placed at 50°C for 10 mins, cooled and acidified with 10μl acetic acid. To the mixture, 0.5mls of water were added and vortexed, followed by 0.5ml hexane, vortexing and centrifugation to focus the interface. The top layer was removed, the hexane extraction repeated, and both supernatants pooled and dried down. Products of FAME reactions were evaluated by analytical TLC, run as for preparative TLC runs above, but using a hexane: diethyl ether: acetic acid (8:2:2) solvent run out for 10 cm, followed by charring with cupric sulfate. The FAME mixture was dissolved in anhydrous dicholoromethane, spiked with a C17 FAME standard (Cayman# 26723) and analyzed by GC/MS on an Agilent 7890B GC coupled to an Agilent 5977A MSD, with an Agilent DB-23 column (60m long, 0.25mm i.d., 0.25μm film thickness), with a staged injection protocol run at 250°C. Separation resolution, identification and reproducibility were assessed using a Supelco 37-FAME standard (cat # CRM47885), and retention times (RTs) were compared to skin sample preparations (**Fig.S1**).

### LC/QTOF-MS mass spectrometric analysis of lipids in sera.

Lipidomic analysis was performed according to Simcox et al^43,44^; briefly, 40μl aliquots of sera were combined with 250μls PBS and 225μls ice-cold MeOH containing internal standards (Avanti Splash cat#3307–07), and homogenized. Samples were then mixed with 750μls of ice-cold MTBE (methyl tert-butyl ether), rehomogenized, and separated by centrifugation (17,000g for 5 mins/4°C). The upper phase was transferred to a new tube, lyophilized and resuspended in 150 μls of isopropanol. Lipids were analyzed by UHPLC/MS/MS in positive and negative ion modes, at a dilution suitable to eliminate saturation issues. Extracts were separated on an Agilent 1260 Infinity II UHPLC system using an Acquity BEH C18 column (Waters 186009453; 1.7 μm 2.1 × 100 mm) maintained at 50 C with VanGuard BEH C18 precolumn (Waters 18003975), using the chromatography gradients described by Jain et al. The UHPLC system was connected to an Agilent 6546 Q-TOF MS dual AJS ESI mass spectrometer and run in both positive and negative modes as described. Samples were injected in a random order and scanned between 100 and 1,500 m/z. Tandem MS was performed at a fixed collision energy of 25 V. The injection volume was 2 μl for positive mode and 5 μl for negative mode.

#### Lipidomic data processing.

Methods for data processing were described by Jain et al^44^. Briefly, MS/MS data were analyzed using Agilent MassHunter Qualitative Analysis and LipidAnnotator for lipid identification^45^. Accuracy of retention times was checked by reference to internal standards. Data was imported into Agilent Profinder for lipid identification and peak integration (using sera-specific libraries). Data were analyzed using MetaboAnalyst free-ware (https://www.metaboanalyst.ca), Microsoft Excel and GraphPad Prism8 software (https://www.graphpad.com/scientific-software/prism).

### Statistical Analysis.

The statistical tests appropriate to each analysis are indicated in Figure legends. To test for normal or lognormal distribution of sample values we used the Anderson-Darling test, outliers were identified using ROUT method. Box-and-whiskers graphs show median values with 10–90 percentile whiskers; other data are expressed as mean ± standard deviation, unless specifically stated.
